# A linear‐transformer hybrid for single‐nucleotide polymorphism‐based genotype‐to‐phenotype prediction in grapevine

**DOI:** 10.1002/tpg2.70335

**Published:** 2026-11-02

**Authors:** Yibin Wang, Murukarthick Jayakodi, Silvas Kirubakaran, Ambika Chandra, Azlan Zahid

**Affiliations:** ^1^ Department of Biological and Agricultural Engineering, TX A&M AgriLife Research Texas A&M University System Dallas Texas USA; ^2^ Department of Soil and Crop Sciences, TX A&M AgriLife Research Texas A&M University System Dallas Texas USA; ^3^ USDA‐ARS, Grape Genetics Research Unit Geneva New York USA

## Abstract

Robust genotype‐to‐phenotype (G2P) prediction is essential for accelerating grapevine (*Vitis* spp.) breeding decisions and genetic gain. However, it remains challenging to measure complex traits under variable field conditions and across years. In this study, we propose a linear‐Transformer (LiT)‐G2P, an automated predictive framework that integrates additive genetic variance effects with Transformer‐based nonlinear interactions using genome‐wide single‐nucleotide polymorphisms (SNPs) data. We evaluated LiT‐G2P on a panel of diverse grape accessions, genotyped with SNP markers and measured for phenotypes across three consecutive years. Target phenotypic traits include leaf hair density and trichome density of grapevines. Across both single‐year and cross‐year testing scenarios, LiT‐G2P consistently improves prediction performance compared with baseline models. For leaf hair density, LiT‐G2P achieved the lowest prediction errors in both single‐year and cross‐year evaluations, with root mean square error (RMSE) values of 0.475 and 0.460 and ± 0.5 accuracies of 78.6% and 72.9%, respectively. For trichome density, LiT‐G2P achieved RMSE values of 0.637 and 0.766 and corresponding accuracies of 62.6% and 58.2%. In addition, we extracted model‐prioritized SNPs from attention weights and applied genotype‐stratified analysis to provide interpretable candidate marker for downstream validation. These results support practical SNP‐based predictive modeling for genomic selection and identify the remaining need for more detailed field data, larger multi‐year grapevine panels, and independent grapevine validation before broader deployment.

## Plain Language Summary

Grapevines have important traits, such as leaf hair and trichome density, that affect plant growth and resilience. Predicting these traits from genetic data are difficult because environmental conditions can change from year to year, making measurements less consistent. This study introduces a new method called genotype‐to‐phenotype (LiT‐G2P) to improve how traits are predicted using genome‐wide single‐nucleotide polymorphism data. The approach is designed to better capture complex relationships between genetic information and observable plant traits. The results show that LiT‐G2P provides more accurate predictions than existing methods in both single‐year and cross‐year tests. It achieved lower prediction errors and strong accuracy for leaf hair density, and it also performed best overall for trichome density. In addition, the model was able to highlight important genetic markers associated with these traits. The study shows that this approach can improve genetic prediction for grapevine breeding. However, more data across

**Abbreviations**
8 HY: interspecific hybrids
AI: artificial intelligence
CapsNet: capsule network
G2P: genotype‐to‐phenotype
GRIN: germplasm resources information network
LiT‐G2P: linear‐transformer genotype‐to‐phenotype
LSTM: long short‐term memory
MAE: mean absolute error
MAPE: mean absolute percentage error
ML: machine learning
MLP: multilayer perceptron
MSE: mean squared error
RMSE: root mean square error
RNN: recurrent neural network
SNP: single‐nucleotide polymorphism
USDA: United States Department of Agriculture
VCF: variant call format
XGBoost: extreme gradient boosting

## 1 INTRODUCTION

Genotype‐to‐phenotype (G2P) relationships are fundamental for advancing agriculture by linking genetic variation to agronomically important traits (Fernandez‐Pozo et al., 2015). Large‐scale genotyping enables the discovery of genetic variants associated with agronomic traits (Bailey‐Serres et al., 2019), disease resistance (Van Esse et al., 2020), and abiotic stress tolerance (Carvalho et al., 2021; Wang et al., 2003), providing a data‐driven basis for selection decisions. Converting G2P associations into reliable predictions of complex phenotypes is essential for accelerating genetic gain in breeding programs, particularly for traits that are quantitative and influenced by environmental interactions. In G2P prediction, genomic inputs are typically encoded as genetic markers single‐nucleotide polymorphisms (SNPs), which represent single‐base DNA variants among individuals (Grinberg et al., 2020). Adopting such genotypic information for phenotype prediction can improve breeding efficiency, accelerate genetic gain, and support the development of resilient cultivars for sustainable agricultural production (Boggess et al., 2013).

In 2024, the United States was the third‐largest grape‐producing country, accounting for approximately 385,000 ha of vineyard area and 6.4 million metric tons of production, representing 5% of global vineyard area and 8% of global grape production (Brief, 2025). Conventional crop breeding in perennial crops such as grapevine (*Vitis* spp.) relies on recurrent selection and evaluation across multiple environments to assemble favorable trait combinations (Eibach & Töpfer, 2015). Although improved cultivars have been produced, these approaches are inherently slow, resource intensive, and often limited in efficiently capturing quantitative traits (Callipo et al., 2025). Wild grapevine species such as *Vitis riparia, V. berlandieri, and V. rupestris* are known to confer abiotic stress tolerance when used as rootstocks, thereby enhancing the stress resilience of cultivated grapevines (Rahemi et al., 2022). However, due to the narrow genetic base of these species (Riaz et al., 2019), it is imperative to identify additional sources of resistance and tolerance to environmental fluctuations through the characterization of beneficial traits, particularly in leaf features. Leaf traits particularly epidermal projections serve as the plant's outermost interface with the environment, mediating complex interactions. Specifically, grapevine leaf hairs and trichomes are epidermal structures that vary among species and cultivars and may contribute to plant identification and protection against biotic and abiotic stresses (Gago et al., 2016; Yang et al., 2024).

Recent advances in phenotyping and next‐generation sequencing transform plant breeding by enabling large‐scale multidimensional data collection (Zhang et al., 2025). This breakthrough is driven by the development of phenotyping systems employing physical, chemical, and biological sensors, supported by emerging artificial intelligence (AI) technologies as well. Phenomics platforms now enable rapid, quantitative assessment of diverse responses, while long‐read sequencing provides high‐resolution genome structure and haplotype diversity (Demidchik et al., 2020). However, extensive genetic diversity and genomic complexity can complicate the dissection of trait architecture (Lappalainen et al., 2024). These challenges are further amplified by environmental effects, which can obscure causal signals and limit transferability. Such genetic and environmental heterogeneity jointly challenges the stability of genotype‐phenotype associations, motivating robust predictive methods that can generalize G2P prediction for effective genomic selection and trait improvement in breeding.

AI and machine learning (ML) methods have advanced G2P modeling by integrating high‐dimensional genomic markers with complex and longitudinal phenotypic observations. Unlike conventional linear approaches, ML models can capture nonlinear genetic effects, epistatic interactions, and structured dependencies to improve explainable predictive performance for polygenic traits and enable data‐driven optimization of selection decisions (John et al., 2022; Novakovsky et al., 2023). A range of deep learning (DL), architectures including recurrent neural networks (RNNs) (Lugo & Hernández, 2021; Quang & Xie, 2016) and long short‐term memory (LSTMs) networks (Tavakoli, 2019) and capsule networks (CapsNet) (Luo et al., 2023), have been explored to learn hierarchical feature representations from genomic data and to model temporal dynamics. Although RNNs and LSTMs can process ordered genomic inputs, their recurrent computation is less efficient for long SNP sequences and may limit the modeling of interactions between widely separated markers. CapsNet can learn hierarchical representations but is primarily designed around local relationships, which are less directly aligned with genome‐wide additive and SNP effects. Transformer‐based architectures adopt self‐attention to model long‐range dependencies and have demonstrated strong utility across bioinformatics tasks, including sequence representation learning, variant effect prediction, and multi‐omics integration (Choi & Lee, 2023). By learning context‐aware embeddings, attention mechanisms in Transformers and genome language models can prioritize informative genomic regions and flexibly aggregate signals across the genome, which fits sparse, heterogeneous, and high‐dimensional breeding data (Consens et al., 2025). Transformer‐based imputation methods employed sequential SNP data for human leukocyte antigen gene analysis (Tanaka et al., 2024). Recent advances in genomic foundation models further illustrate the potential of large‐scale pretraining for transferable biological representations. DNABERT introduced bidirectional language modeling for DNA and learned generalizable sequence features from upstream and downstream nucleotide context (Ji et al., 2021). HyenaDNA expanded efficient long‐context modeling to support single‐nucleotide resolution with context windows reaching up to one million tokens, enabling representation learning over gene‐scale and genome‐scale spans (Nguyen et al., 2023). Similarly, the Nucleotide Transformer scaled model capacity to billions of parameters and leveraged training corpora spanning thousands of human genomes and hundreds of genomes across diverse species, improving cross‐task transfer and robustness (Dalla‐Torre et al., 2025). Despite these advances, predictive breeding frameworks that jointly utilize genomics, phenomics, and AI remain underdeveloped in agriculture crop plants. Moreover, while many genomic foundation models are trained on full DNA sequences, most applied breeding programs rely on cost‐effective SNP genotyping (Mammadov et al., 2012), resulting in a gap between sequence‐level representation learning and marker‐based G2P prediction.

To address this gap, we propose an AI‐empowered predictive breeding framework, Linear‐Transformer Genotype‐to‐Phenotype (LiT‐G2P) that employs genome‐wide SNP variants to support multi‐trait improvement in grapevine. Building on our proposed LiT‐G2P model, the predictor decomposes genetic effects into (1) additive main effects captured by a linear component and (2) nonlinear interactions modeled by a Transformer‐based component, enabling simultaneous learning of interpretable marker contributions and dependencies across the genome. We focus on two related traits, hair density and trichome, formulating the prediction as a multi‐trait learning problem with a shared LiT backbone and a two‐dimensional output layer. This design allows the model to exploit shared genetic architecture and phenotypic correlation between traits, effectively adopting shared information in the high‐dimensional, low‐sample regime (N≪p). The linear main effect branch and Transformer interaction branch are trained across traits, while only the final output heads are trait specific. Such parameter sharing enables the backbone to learn genomic representations that are informative for both traits, acting as an additional form of regularization to improve robustness. Large genomic foundation models generally require substantially larger training populations than the 320 accessions available here. LiT‐G2P was therefore designed as a compact, task‐specific architecture rather than a large‐scale pretrained Transformer. Non‐overlapping SNP patches reduce the input sequence, while the parallel linear branch provides an explicit additive‐effect inductive bias. The Transformer component complements this additive representation by modeling dependencies among genomic regions. Nevertheless, given the high marker‐to‐accession ratio, the findings should be considered specific to the evaluated panel until validated in larger and independent grapevine populations. Beyond grapevine, the proposed framework provides a practical template for integrating SNP‐based G2P prediction into breeding pipelines for other crops where conventional selection is limited or inefficient. The main contributions of this study are presented as:

1. a grapevine‐focused SNP‐based AI framework LiT‐G2P that unifies linear additive main effects and nonlinear interaction Transformer‐based modeling for G2P prediction, reflecting the genetic basis of complex traits;
2. a multi‐trait formulation on SNP markers that jointly predict target traits using a shared backbone and provides interpretability by selecting top‐ranked SNPs and validating via genotype‐stratified phenotype analysis;
3. a within‐panel evaluation against representative baselines on a grape SNP dataset to quantify the benefits of interaction modeling and multi‐trait learning, demonstrating improved single‐year and cross‐year prediction for studied traits.

## 2 MATERIALS AND METHODS

### 2.1 Data acquisition

The National Clonal Germplasm Repository for Grape was established in 1984 in Geneva, New York. The grape repository consists of 1177 unique accessions of *Vitis* species, including a large collection of cold‐tolerant *Vitis* hybrids (HY) and a wide range of *Vitis* species native to cooler climates; most are native to North America, but some accessions originated in Europe and Asia. To establish feasibility, we selected a diversity panel of 320 grape (*Vitis* spp.) accessions to capture broad genetic variation representative of breeding‐relevant germplasm. Vines are managed following standard commercial viticultural practices, including routine weed and pest control, fertilization, and winter dormant pruning conducted between February and March. The vineyard is non‑irrigated due to sufficient natural rainfall. Most accessions are maintained as two self‑rooted, side‑by‑side replicated vines. The genus *Vitis* comprises 68 recognized species, and the Geneva *Vitis* repository maintains more than 16 of these species along with over 700 interspecific HYs derived from *Vitis vinifera* and multiple North American wild species. The 200 HYs included in our study were generated from diverse wild genetic backgrounds. The diversity panel comprised 200 HY, 69 *V. riparia* accessions (RI), and 51 accessions representing additional wild and cultivated *Vitis* groups. The HYs and wild accessions capture genetic backgrounds relevant to cold tolerance and resistance to diseases and pests and represent germplasm of potential value for grapevine improvement (Gutierrez et al., 2021; Herniter et al., 2026).

Leaf discs were collected from over *Vitis* germplasm accessions, including *Acerifolia‐*8 accessions, *Cineria‐*4 accessions, *Hybrid‐*200 accessions, *Labrusca‐*2 accessions, *Palmata‐*3 accessions, *Riparia‐*69 accessions, *Rupestris‐*18 accessions, *Vitis sp‐*8 accessions, *V. vinifera* ssp*. Vinifera‐*3 accessions, *Vitis x champinii‐*1 accession, and *Vulpina‐*4 accessions in the United States Department of Agriculture (USDA) in Geneva, NY. The accessions were sampled in summers of 2022, 2023, and 2024 to evaluate density of leaf hair and trichome (Table S1). Baseline phenotypic measurements for the 320 accessions were collected during summer 2022 for 129 accessions and summer 2023 for 191 accessions. The 2024 phenotype dataset remained unchanged and served as the target dataset. Accordingly, single‐year evaluation was conducted within the 2024 dataset, whereas cross‐year evaluation used the combined 2022–2023 baseline dataset for training and the 2024 dataset for testing. Following quality control and filtering, 15,388 SNP markers were retained for each accession and used as inputs for model development. The markers were distributed across all 19 grapevine chromosomes, with the number per chromosome ranging from 571 on chromosome 15 to 1203 on chromosome 14.

The vineyard is located at an elevation of approximately altitude of 200 m above sea level in USDA plant hardiness zone 6b (characterized by average annual extreme minimum temperatures of −20.6°C to −17.8°C) (https://planthardiness.ars.usda.gov/). Most accessions were represented as two replicates of self‐rooted vines. Taxonomic identification was determined through accession passport records and the National Plant Germplasm System database. The botanical nomenclature of all accessions evaluated in this study can be queried in the germplasm resources information network (GRIN)‐Global Database (https://www.ars‐grin.gov/).

For the leaf appendage measurements collected in 2022–2024 (38‐year‐old to 40‐year‐old vines), the fully matured and physiologically active third leaf from the top of two vines representing each accession was collected in the month of July (Table S1). Specifically, the third fully expanded leaf from the shoot apex was collected from each of the two vines representing every accession. This standardized developmental stage ensures consistency in physiological status across accessions and enhances the reproducibility and interpretability of the resulting measurements. From each leaf collected from two different vines, four 1‐cm discs per leaf were subsampled and placed in a balanced lattice design and arrayed on eight 32 × 26 × 2 cm Pyrex glass trays (Corning, NY) containing 1% agar medium. A grid adhered under each tray was used to guide uniform sample placement in a complete replicate block design for imaging with the BlackBird robot. The information on the BlackBird, a high‐throughput phenotyping device used in this study, is explained in detail in Sapkota et al. (2025). In this study, we investigated two biologically related phenotypic traits, leaf hair density and trichome density. Figure 1 provides an overview of the phenotyping workflow and trait variability, illustrating BlackBird microscopic imaging of grapevine leaf discs for quantifying leaf hair and trichome density and representative leaf discs from diverse grape accessions highlighting broad phenotypic variation. The studied traits are relevant to plant protection and stress responses and can exhibit substantial genetic control while remaining sensitive to environmental conditions (Gago et al., 2016; Yang et al., 2024). Phenotypes were then evaluated and recorded to capture realistic variation arising from both genotype and environment. Each trait was manually scored from the high‐magnification BlackBird images with an ordinal scale from 0 to 3 in increments of 0.25, providing fine‐grained resolution for quantitative modeling and enabling evaluation of predictive performance across years and traits.

**FIGURE 1:**
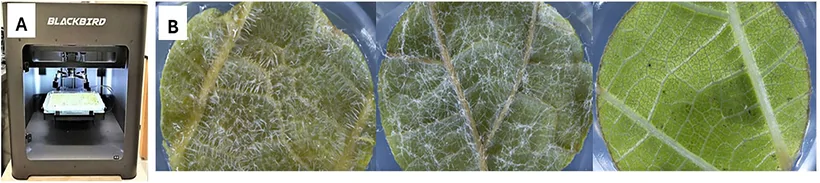
(A) BlackBird microscopic imaging of grapevine leaf discs to quantify leaf hair and trichome density; (B) Leaf discs of diverse grape accessions showing phenotypic variability for leaf hair and trichome density.

With the blackbird outpacing manual microscopic image of leaf disc up to 60‐fold, two imaginary axial transects (horizontal and vertical) were viewed across each leaf disc at 200× magnification that enabled the manual scoring of trichome density scored as 0 (no trichome), 1 (sparse trichomes along the main leaf veins), 2 (regular trichomes along the main leaf veins), and 3 (regular trichomes widespread across the leaf), whereas the leaf hair density is scored as 0 (no hair), 1 (sparsely covered in hair with 75+% of the leaf surface visible), 2 (covered in hair but with 25%–50% of the leaf surface still visible) and 3 (completely covered in thick hair, nearly completely white with little to no leaf surface visible). As there is a lack of established standardized quantitative methods for grapevine leaf hair and trichome density that are not yet available to ensure reliability of the phenotype data used for G2P prediction, all trichome and leaf hair density measurements were instead manually quantified. This was feasible because the BlackBird imaging platform enabled acquisition of high‑magnification microscopy images (60×; 8000 × 5000 pixels per cm), allowing precise manual scoring of both traits. As standardized quantitative methods for assessing grapevine leaf hair and trichome density are not yet available, we acknowledge that this visual scoring approach is a limitation and emphasize the need to develop automated methods to precisely quantify trichome and leaf hair density based on percent area estimation. Figure 2 illustrates the phenotypic scoring criteria for the two target traits, showing representative examples of leaf hair density and trichome density.

**FIGURE 2:**
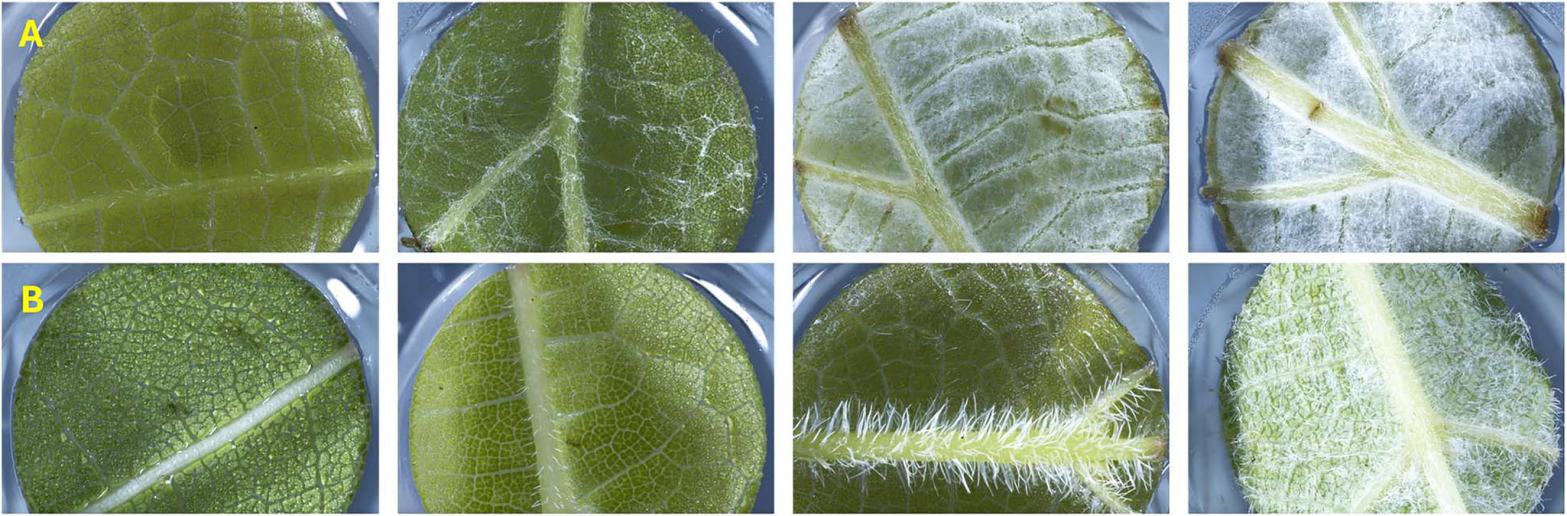
Phenotypic variability observed for leaf hair (A) and trichome (B) densities were scored as 0, 1, 2, and 3 from left to right.

Genotypic data were generated using a high‐density SNP array, producing 15,388 variants after standard quality control and filtering. In particular, SNPs and accessions were screened for overall call rate to remove unreliable genotyped markers and samples. Markers with low minor allele frequency of 0.05 were also excluded to reduce noise and improve statistical stability in the downstream modeling. We further filtered missing SNPs (6.45%) and removed variants with ambiguous allele coding or inconsistent genotype clustering. The resulting SNP set was curated, aligned across accessions, and formatted as a consistent genotype call/matrix (0/1/2) suitable for sequential modeling and cross‐year evaluation. Following conversion of genotype calls to additive dosages of 0, 1, and 2, all 317,589 missing calls, representing 6.45% of the genotype matrix, were uniformly assigned a dosage of 0 corresponding to the homozygous reference genotype. No additional or conditional imputation criteria were applied. Dosage 0 was the marker‐wise modal genotype at 14,458 of the 15,388 SNPs and matched marker‐wise modal imputation for 94.4% of missing calls. The remaining 17,702 differing calls represented 0.36% of the complete genotype matrix.

### 2.2 Data preprocessing

For data preparation, we first processed Variant Call Format (VCF) genotype calls into SNP matrices and aligned them with phenotypic records using accession identifiers and year‐specific observations. Genotypes were converted to alleles (0/1/2) to provide a consistent numerical representation for downstream modeling, where 0/0 refers to homozygous reference allele as 0, 0/1 or 1/0 refers to heterozygous allele as 1, and 1/1 refers to homozygous alternate allele as 2, respectively. This encoding yields an N×L matrix, where N denotes the total number of accessions and L is the number of SNP markers retained after quality control. Remaining missing genotype calls (6.45%) were imputed as major genotype to ensure a complete and consistent input matrix so that all accessions shared the same marker set and dimensionality. Phenotypic measurements were standardized to match the corresponding genotype entries, and records with missing trait scores were excluded from supervised training and evaluation. To model genome‐wide dependencies beyond additive effects, we further implemented a Transformer‐based architecture to capture both local and global linkage patterns across the genome. SNPs were grouped into non‐overlapping patches of 64 SNPs with each patch embedded into a 128‐dimensional latent space. Positional encodings were added to preserve chromosomal order across patches. This patch tokenization allows the proposed LiT‐G2P to learn hierarchical SNP patterns while maintaining tractable attention computation over genomic inputs.

### 2.3 Methodology

#### 2.3.1 The proposed LiT‐G2P predictor

Hereby, we detail the proposed LiT‐G2P framework for phenotypic trait prediction of grape leaf hair and trichome density from SNP data. This HY architecture decomposes phenotype prediction into additive main effects and nonlinear interaction effects. Given an accession i, the model takes as input the SNP vector $x_{i}\in R^{L}$, where the full genotype dataset can be represented by the matrix $X\in R^{N\times L}$. N denotes the number of accessions and L denotes the number of SNP variants. Each entry $X_{i,j}\in \left\{0,1,2\right\}$ corresponds to the allele dosage at SNP marker j for individual i. The output Y of the model is represented by the phenotype vector $y_{i}$ as:

(1) $$y_{i}=\left[\begin{array}{c} y_{i}^{hair}\\ y_{i}^{trich} \end{array}\right]\;\in R^{2}$$

The overall phenotype prediction ${\hat{y}}_{i}$ is modeled as:

(2) $${\hat{y}}_{i}=W_{\mathrm{main}}\;x_{i}+f_{TF}\left(x_{i}\right)$$

$W_{\mathrm{main}}\in R^{2\times L}$ are the additive SNP effects that describe the independent contribution of each gene's alleles to a trait. $f_{TF}\left(\cdot \right)$ is the Transformer‐based interaction that learns epistasis interaction where the effect of one gene is dependent on the presence of one or more other genes. These two complementary components provide a biologically grounded decomposition of genetic architecture by combining interpretable main effects with higher‐order interactions to better reflect how complex traits arise from genomic contributions. Specifically, the linear main effect branch provides a direct additive mapping from SNP dosages to trait values by:

(3) $$\;{\hat{y}}_{i}^{\mathrm{main}}=W_{0}x_{i}$$

This branch models independent marker contributions and serves as a strong, interpretable baseline within the HY model. In the proposed method, $W_{0}$ is the weight matrix of a trainable linear layer, with one row per trait and one column per SNP. It is not estimated in a separate genome‐wide association study. Instead, $W_{0}$ and its bias are optimized jointly with the Transformer branch by back‐propagation under the multi‐trait mean squared error (MSE) loss. The optimizer updates $W_{0}$ in every minibatch, and weight decay provides L2 regularization. Thus, the linear branch contributes a directly interpretable additive dosage effect while remaining calibrated to the nonlinear interaction branch.

In the Transformer interaction branch, each SNP genotype value $X_{i,j}\in \left\{0,1,2\right\}$ is mapped into a continuous embedding as:

(4) $$e_{i,j}=\mathrm{Embed}\left(X_{i,j}\right)\in R^{D}$$

The embeddings are stacked across all variants yielding:

(5) $$E_{i}=\left[e_{i,1},e_{i,2},\text{…},e_{i,L}\right]\in R^{D\times L}$$

where D refers to the embedding hidden dimension. To reduce the sequence length and learn local SNP patterns, variants are grouped into non‐overlapping patches of size P. Each genotype dosage $x_{i}$ is input as a categorical token and passed through an embedding lookup $E_{i}$. The embedded sequence is transposed to channels‐first form and passed through a depthwise one‐dimensional convolution with kernel size and stride equal to the patch size. Each convolutional channel summarizes local dosage patterns within a 64‐SNP window, after which sinusoidal positional encodings are added. This design keeps attention computationally tractable for markers while the parallel linear branch still uses the complete marker vector. A depthwise 1D convolution produces patch tokens by:

(6) $$Z_{i}=\left[z_{i,1},z_{i,2},\text{…},z_{i,L^{\prime }}\right]\;\in R^{D\times L^{\prime }}$$

where $L^{\prime }=L/P$ is the number of patches given a patch size P. To preserve marker order across patches, sinusoidal positional embeddings $p_{q}$ are applied by:

(7) $${\tilde{z}}_{i,q}=z_{i,q}+p_{q}$$

where $p_{q}$ is the sinusoidal positional embedding for token *q* in $L^{\prime }$. A standard Transformer encoder stack then models long‐range dependencies among patches:

(8) $$h_{i,q}=\mathrm{TransformerBlock}\left({\tilde{z}}_{i,1},\;{\tilde{z}}_{i,2},\text{…},{\tilde{z}}_{i,L^{\prime }}\right)$$

Patch‐wise hidden states are aggregated into a single sequence representation with mean pooling as:

(9) $${\bar{h}}_{i}=\frac{1}{L^{\prime }}\;\sum_{q=1}^{L^{\prime }}h_{i,q}\in R^{D}$$

The pooled representation is mapped to trait prediction using a multilayer perceptron (MLP) head by:

(10) $${\hat{y}}_{i}^{\mathrm{inter}}=W_{2}\;\sigma \left(W_{1}{\bar{h}}_{i}\left(x_{i}\right)+b_{1}\right)+b_{2}$$

where $W_{1}$ and $W_{2}$ are trainable weight matrices, $b_{1}$ and $b_{2}$ are biases, and σ(·) is a pointwise nonlinearity. The resulting ${\hat{y}}_{i}^{\mathrm{inter}}$ captures nonlinear interaction‐driven genetic effects. Therefore, the overall prediction can be represented as:

(11) $${\hat{y}}_{i}=W_{0}\;x_{i}+W_{2}\sigma \left(W_{1}{\bar{h}}_{i}\left(x_{i}\right)+b_{1}\right)+b_{2}$$

LiT‐G2P performs joint prediction of hair density and trichome score with a shared backbone consisting of linear and Transformer branches. In implementation, the model outputs a two‐dimensional vector $y_{i}\in R^{2}$ through a shared MLP head with two output units, allowing joint learning across traits and improving robustness N≪p. Model parameters are optimized using MSE over the two traits by the loss L as:

(12) $$\begin{array}{ccc} L & = & \frac{1}{N}\;\sum_{i=1}^{N}{∥{\hat{y}}_{i}-y_{i}∥}_{2}^{2}=\frac{1}{N}\;\sum_{i=1}^{N}\left[{\left({\hat{y}}_{i}^{\mathrm{hair}}-y_{i}^{\mathrm{hair}}\right)}^{2}\right.\\ & & \left.+\;{\left({\hat{y}}_{i}^{\mathrm{trich}}-y_{i}^{\mathrm{trich}}\right)}^{2}\right] \end{array}$$

Regarding the model configuration and regularization, the encoder stack consists of 2 Transformer encoder layers with four self‐attention heads per layer, and a feedforward network of 256 hidden units. This design provides sufficient hierarchical abstraction of local‐to‐global SNP patterns while maintaining computational efficiency and stable convergence during training. The encoder employs multi‐head self‐attention to model dependencies among SNPs. Thus, the network can weigh both short‐range and long‐range interactions. Regularization was applied with a dropout rate of 0.2 and layer normalization. The final sequence representation was aggregated using a class token followed by a linear projection layer to predict trait values. The training loop used minibatches of 16 accessions with 4 attention heads, a maximum of 300 epochs, and patience‐based stopping to reduce overfitting in the small‐sample setting. The final LiT‐G2P configuration used the Adam optimizer with a learning rate of 5 × 10^−4^ and weight decay of 10^−5^. The end‐to‐end workflow of LiT‐G2P is outlined in Algorithm 1.

ALGORITHM 1 **The proposed LiT‐G2P predictor.**

**Table:** 

**Input**: Genotype matrix X with entries $X_{i,j}$; Phenotype matrix Y with vector $y_{i}$; Patch size P; Embedding dimension D; Number of accessions N; Number of SNP variants L. **Parameters**: $W_{0}$; Embed (·); Positional embeddings $p_{q}$; Transformer encoder TF(·); MLP head $\left(W_{1},W_{2},b_{1},b_{2}\right)$	
**start**	
	$L^{\prime }\leftarrow L/P$
	**for** i=1 to N **do**
	$x_{i}\leftarrow X_{i,:}^{⊤}\in R^{L}$
	Main effects: $\;{\hat{y}}_{i}^{main}=W_{0}x_{i}$
	**for** j=1 to L **do**
	$e_{i,j}=\mathrm{Embed}\left(X_{i,j}\right)$
	**end for**
	$E_{i}=\left[e_{i,1},e_{i,2},\text{…},e_{i,L}\right]$ $Z_{i}=\left[z_{i,1},z_{i,2},\text{…},z_{i,L^{\prime }}\right]$ **for** q=1 to $L^{\prime }$ **do**
	${\tilde{z}}_{i,q}=z_{i,q}+p_{q}$
	**end for**
	$h_{i,q}=\mathrm{TF}\left({\tilde{z}}_{i,1},\;{\tilde{z}}_{i,2},\text{…},{\tilde{z}}_{i,L^{\prime }}\right)$
	Pooling: ${\bar{h}}_{i}=\frac{1}{L^{\prime }}\;\sum_{q=1}^{L^{\prime }}h_{i,q}$ $\;{\hat{y}}_{i}^{inter}=W_{2}\;\sigma \left(W_{1}{\bar{h}}_{i}\left(x_{i}\right)+b_{1}\right)+b_{2}$ **output** Final prediction: ${\hat{y}}_{i}$
	**end for** $Y\leftarrow {\left[{\hat{y}}_{1}^{⊤},{\hat{y}}_{2}^{⊤},\text{…},{\hat{y}}_{N}^{⊤}\right]}^{⊤}$
**end**

#### 2.3.2 Performance evaluation

Since the target phenotypes are ordinal density scores but the expected breeding use is ranking and prioritizing accessions, we managed prediction as a continuous regression task and evaluated both error magnitude and decision‐level tolerance. Initial experiments with lightweight LiT‐G2P demonstrated feasibility but were limited by small sample size and underfitting. By computing allele‐specific embeddings directly from the VCF data, we generated biologically meaningful feature representations without requiring whole‐genome sequences. These embeddings were aggregated across the genome and coupled with regularized regression to provide robust trait prediction under limited sample sizes. We trained the LiT‐G2P with MSE loss for continuous traits and monitored performance with root mean square error (RMSE), mean absolute error (MAE), and mean absolute percentage error (MAPE). In addition to conventional performance metrics, we evaluated categorical accuracy within error thresholds of 0.5 units. This bin‐based approach reflects practical breeding utility, where predictions that fall within half a unit of the observed phenotype are still considered actionable for selection. Consequently, we assessed model performance not only in terms of statistical fit but also in terms of decision‐making reliability for downstream breeding applications.

Evaluation was designed around grapevine breeding use rather than external benchmarking. The same‐year protocol used a random within‐season 80%/20% train/test split to estimate performance when training and deployment phenotypes come from the same growing season. Model performance was evaluated using fivefold cross‐validation, with accession‐level grouping maintained across folds to prevent data leakage and ensure that all observations from the same accession remained within the same training or testing partition. Measurements from the two vine replicates were aggregated to obtain one accession‐level phenotype value for each trait and year before G2P alignment. All data splits were subsequently performed at the accession level using unique accession identifiers. The same partition was maintained across traits, years, and models, ensuring that neither vine‐level replicates nor observations from the same accession appeared in both the training and testing sets. The cross‐year protocol trained models using phenotypes from one season and evaluated them against held‐out accessions measured in the other season, quantifying season‐to‐season transfer within the grapevine panel. This protocol approximates applying genomic predictions when a model trained from one phenotyping season is used to support selection decisions in a later or alternate season. We evaluate the prediction performance of the proposed method against the baseline G2P prediction methods, including linear ridge regression, extreme gradient boosting (XGBoost), and baseline Transformer, with the same SNP embedding and patch encoder but without the additive linear branch. Relevant performance metrics are calculated in Equations (13)–(16) as:

(13) $$\mathrm{RMSE}\left({\hat{y}}_{i},\;y_{i}\right)=\sqrt{\frac{1}{N}\sum_{i=1}^{N}{\left(y_{i}-{\hat{y}}_{i}\right)}^{2}}\;$$

(14) $$\mathrm{MAE}\left({\hat{y}}_{i},\;y_{i}\right)=\frac{1}{N}\;\sum_{i=1}^{N}\left|y_{i}-{\hat{y}}_{i}\right|$$

(15) $$\mathrm{MAPE}\left({\hat{y}}_{i},\;y_{i}\right)=\frac{1}{N}\;\sum_{i=1}^{N}\left|\frac{y_{i}-{\hat{y}}_{i}}{y_{i}+\varepsilon }\right|$$

(16) $$\mathrm{AC}C_{\pm 0.5}\;\left({\hat{y}}_{i},\;y_{i}\right)=\frac{1}{N}\;\sum_{i=1}^{N}1\left(\left|y_{i}-{\hat{y}}_{i}\right|\leq 0.5\right)$$

where $y_{i}$ and ${\hat{y}}_{i}$ refer to the actual and predicted plant phenotypic trait vectors, respectively. ${\bar{y}}_{i}$ is the sample mean, and ε refers to the constant to avoid division by zero in MAPE. In addition to plant‐wise prediction, we also evaluate the model at the population level by comparing the predicted and observed trait‐score distributions across accessions, assessing whether the model preserves overall phenotypic variability and ranking patterns.

To improve interpretability, we extracted the Top‐*k* informative SNPs prioritized by the LiT‐G2P attention mechanism. Because the Transformer operates on patch tokens, we first aggregated attention weights across heads and layers to obtain a single importance score per token. Specifically, let $A^{\left(m,h\right)}$ denote the self‐attention matrix from layer m and head h. A patch‐level relevance score is computed by averaging attention across heads and layers to define the importance score $s_{k}$ as,

(17) $$s_{k}=\frac{1}{MH}\;\sum_{m=1}^{M}\sum_{h=1}^{H}A_{q,k}^{\left(m,h\right)}$$

This SNP importance can therefore be quantified by aggregating patch‐level attention weights across layers and heads, mapping each SNP to its corresponding patch score. In evaluation, we rank and report the top‐*k* informative SNPs according to the main‐effect weights for the target traits, highlighting markers with the strongest estimated genetic effects. The resulting SNP list should be interpreted as model prioritization rather than causal evidence. We use it to nominate markers for genotype‐stratified phenotype summaries, nearby‐gene inspection, and future biological validation in grapevine.

## 3 RESULTS AND DISCUSSION

Performance of single‐year and cross‐year prediction is shown in Tables 1, 2 for leaf hair density and trichome density across different model families. Across both traits, single‐year evaluation targeted on test year 2024 yields consistently better performance than cross‐year testing, indicating that year‐to‐year variation introduces a meaningful distribution shift in the G2P mapping. This effect is especially evident for the classical baselines that linear ridge and XGBoost show notable degradations in cross‐year accuracy; for example, hair density $\mathrm{AC}C_{\pm 0.5}$ drops to 50 to 52%, suggesting limited ability to generalize when phenotypic expression changes across seasons. In contrast, Transformer‐based models exhibit improved resilience under cross‐year evaluation, maintaining substantially lower error and higher tolerance accuracy, consistent with their capacity to learn distributed, nonlinear dependencies among SNP markers. Genomic relatedness was estimated using the VanRaden method based on polymorphic SNPs retained from the post‐imputation genotype matrix. The mean relationship among distinct accessions was −0.003. Mean within‐group relationships were 0.399 for *V. riparia*, 0.065 for HYs, and 0.093 for the remaining genetic groups, whereas the mean relationship between HYs and *V. riparia* was −0.160. These results confirmed substantial within‐group relatedness and population structure in the diversity panel. SNP‐based principal component analysis revealed population structure within the diversity panel (Figure 3). PC1, PC2, and PC3 explained 10.9%, 5.6%, and 4.2% of the genomic variation, respectively. *Vitis riparia* accessions clustered predominantly along the positive direction of PC1, whereas the HYs were more broadly distributed and partially overlapped with the other Vitis groups. These patterns demonstrate the genetic heterogeneity of the panel and support accounting for population structure during model development and evaluation.

**TABLE 1:** Comparison of prediction performance for leaf hair density under single‐year and cross‐year.

Testing scenario	Method	RMSE	MAE	MAPE	$\mathrm{AC}C_{\pm 0.5}$
**Single‐year**	Linear Ridge	0.762 ± 0.060	0.492 ± 0.044	47.8 ± 3.6%	64.5 ± 7.3%
	XGBoost	0.738 ± 0.048	0.478 ± 0.031	43.9 ± 4.7%	65.1 ± 4.5%
	Transformer	0.624 ± 0.062	0.392 ± 0.045	30.1 ± 8.9%	79.7 ± 4.5%
	LiT‐G2P (ours)	0.475 ± 0.045	0.371 ± 0.028	27.2 ± 6.2%	78.6 ± 4.3%
**Cross‐year**	Linear Ridge	0.803 ± 0.058	0.603 ± 0.037	58.6 ± 8.4%	48.7 ± 7.4%
	XGBoost	0.772 ± 0.046	0.595 ± 0.030	51.9 ± 4.4%	51.6 ± 6.8%
	Transformer	0.658 ± 0.072	0.526 ± 0.057	36.1 ± 6.7%	68.7 ± 5.9%
	LiT‐G2P (ours)	0.460 ± 0.056	0.401 ± 0.045	32.4 ± 7.2%	72.9 ± 5.5%

Abbreviations: MAE, mean absolute error; MAPE, mean absolute percentage error; RMSE, root mean square error; XGBoost, extreme gradient boosting.

**TABLE 2:** Comparison of prediction performance for trichome density under single‐year and cross‐year.

Testing scenario	Method	RMSE	MAE	MAPE	$\mathrm{AC}C_{\pm 0.5}$
**Single‐year**	Linear Ridge	0.933 ± 0.042	0.778 ± 0.035	53.8 ± 9.3%	51.1 ± 3.7%
	XGBoost	0.760 ± 0.028	0.591 ± 0.031	51.7 ± 8.4%	56.9 ± 2.9%
	Transformer	0.630 ± 0.056	0.588 ± 0.045	44.3 ± 8.7%	59.2 ± 4.8%
	LiT‐G2P (ours)	0.637 ± 0.029	0.562 ± 0.039	44.2 ± 8.5%	62.6 ± 3.4%
**Cross‐year**	Linear Ridge	0.982 ± 0.045	0.757 ± 0.032	59.0 ± 7.5%	44.8 ± 5.0%
	XGBoost	0.921 ± 0.049	0.746 ± 0.039	55.1 ± 7.0%	50.1 ± 2.4%
	Transformer	0.815 ± 0.121	0.589 ± 0.109	49.2 ± 7.3%	50.7 ± 5.7%
	LiT‐G2P (ours)	0.766 ± 0.050	0.560 ± 0.049	45.9 ± 6.6%	58.2 ± 4.2%

Abbreviations: MAE, mean absolute error; MAPE, mean absolute percentage error; RMSE, root mean square error; XGBoost, extreme gradient boosting.

**FIGURE 3:**
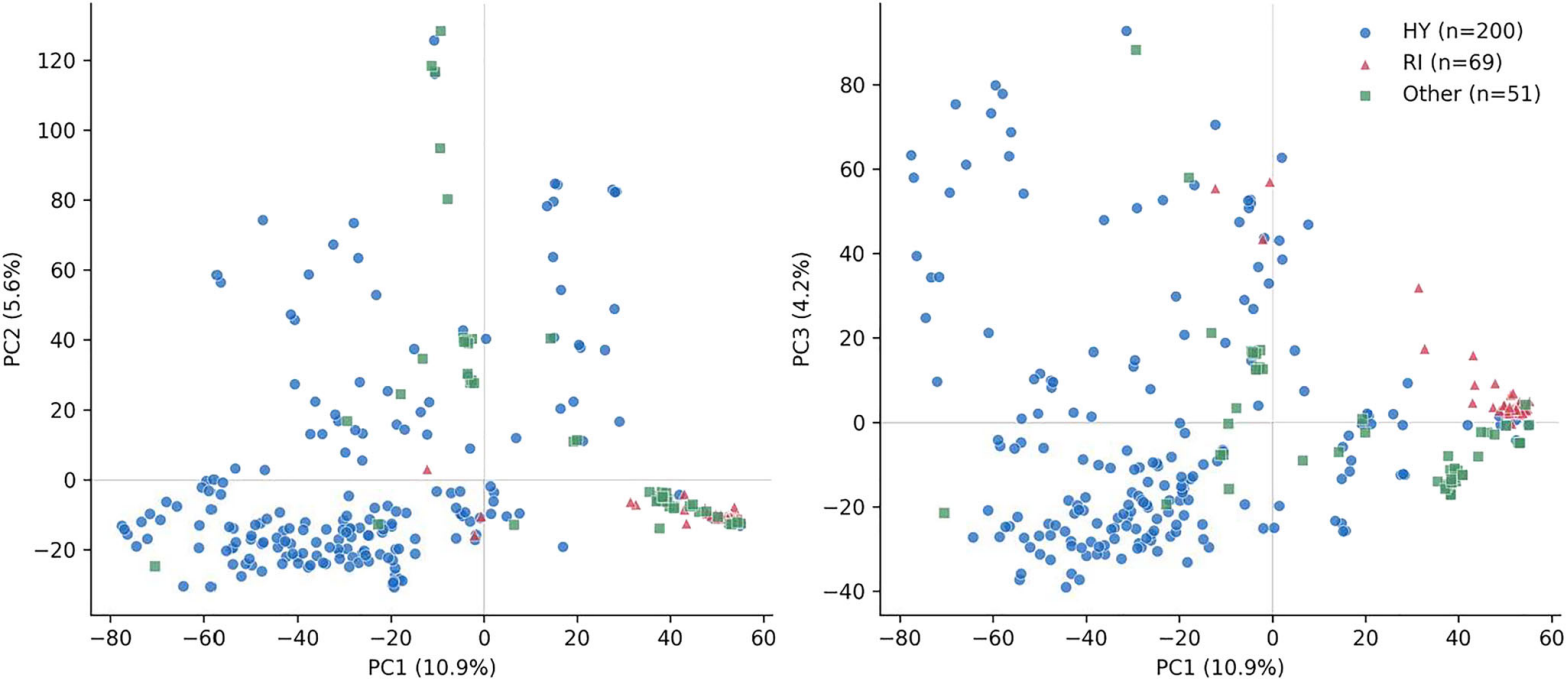
Single‐nucleotide polymorphism (SNP)‐based principal component analysis (PCA) of the 320‐accession grapevine diversity panel.

For hair density prediction according to Table 1, LiT‐G2P achieves the strongest overall performance in both scenarios, with the lowest RMSE and MAPE in single‐year testing of 0.475% and 27.2%, respectively. The proposed method also remains the least cross‐year degradation with 0.460 cross‐year RMSE and 72.9% $\mathrm{AC}C_{\pm 0.5}$. Notably, the baseline Transformer improves over ridge and XGBoost, yet LiT‐G2P provides an additional improvement, supporting the benefit of decomposing genetic effects into additive main effects plus Transformer‐modeled interactions. While the Transformer attains slightly higher accuracy than LiT‐G2P in the single‐year case, LiT‐G2P reduces continuous prediction error more strongly, that is, lower RMSE, MAE, and MAPE, indicating less deviations and better calibration even when small threshold boundary shifts affect the categorical tolerance metric.

Trichome density prediction performance is uniformly lower than for hair density, as demonstrated in Table 2. This indicates the increasing difficulty of this trait, potentially due to stronger environmental sensitivity, measurement variability, or a more complex genetic architecture. Nevertheless, LiT‐G2P presents the best prediction performance under both evaluation regimes, outperforming the baseline Transformer and the classical methods in RMSE, MAE, and accuracy. Furthermore, cross‐year robustness is improved with an RMSE of 0.766 and $\mathrm{AC}C_{\pm 0.5}$ of 58.2%. Collectively, these results suggest that the proposed LiT‐G2P framework provides a practical advantage for breeding‐oriented prediction, particularly when generalization across years is required, by combining stable additive signals with flexible modeling of dependencies in high‐dimensional SNP data.

Nonparametric statistical tests were further performed to identify significant overall differences among the four models for single‐year leaf hair density (*p* = 0.005), single‐year trichome density (*p* = 0.012), and cross‐year leaf hair density (*p* = 0.003). No significant overall difference was detected for cross‐year trichome density (*p* = 0.333). In addition, LiT‐G2P showed significantly lower absolute error than the conventional Transformer for cross‐year trichome prediction (*p* = 0.041), while differences between these models in the other evaluation settings were not statistically significant. Nevertheless, the modeling dataset contained accession‐level annual averages. Consequently, genotype × year variance could not be separated reliably from within‐accession residual variation, and homogeneity of vine‐level residual variances could not be formally assessed.

The impact of cross‐year evaluation on G2P prediction is summarized in Figure 4 by plotting the change in performance relative to single‐year testing (Δ = cross‐year − single‐year) for two target traits. Positive values for RMSE/MAE/MAPE indicate increased prediction error under cross‐year transfer, while negative values for accuracy indicate reduced classification‐style agreement, jointly visualizing the robustness gap around a zero baseline. Across both traits, conventional baselines show larger error inflation and accuracy degradation. Transformer‐based models achieve improved cross‐year stability, with LiT‐G2P consistently producing smaller error increases and accuracy drops, suggesting that representation learning better captures year‐stable genomic signals and mitigates distribution shift. The presented layout provides an overall comparison of cross‐year generalization and highlights that the proposed LiT‐G2P most effectively preserves predictive performance across years.

**FIGURE 4:**
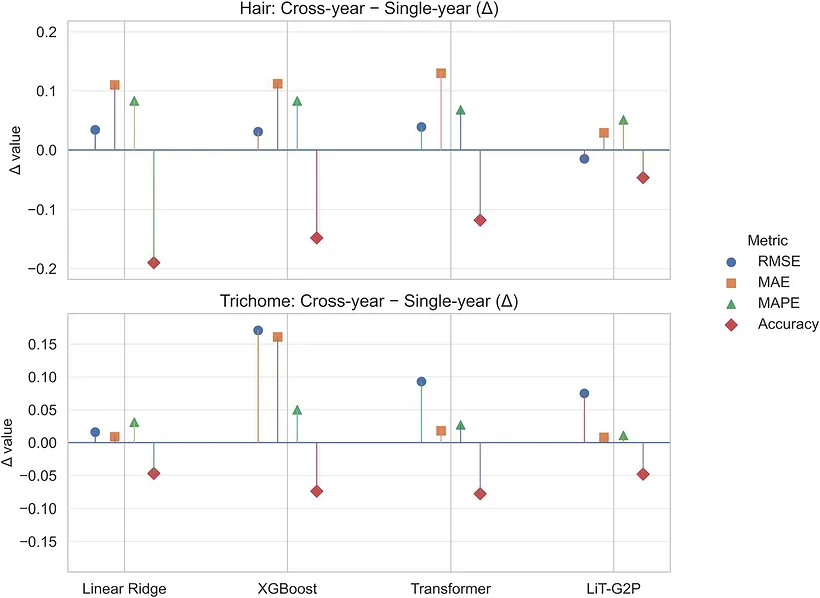
Diverging generalization shift from single‐year to cross‐year prediction: error increases (*Δ* > 0) and accuracy decreases (Δ < 0) across models and traits.

A key limitation revealed in the current analysis is that predictive performance degrades under cross‐year evaluation, indicating sensitivity to environmental shifts in phenotypic expression and measurement conditions. Although LiT‐G2P and the baseline Transformer generalize better than linear ridge and tree models, both still show increased error and reduced accuracy when models trained on one season are applied to another, consistent with genotype‐by‐environment effects and potential differences in field microclimate, management, or imaging conditions across years. This limitation is particularly observed for trichome density, where overall errors remain higher, and tolerance accuracy is lower than hair density in both single‐year and cross‐year settings.

To assess the influence of genetic‐group imbalance, we conducted a population‐aware sensitivity analysis using SNP‐derived principal components and a group‐stratified held‐out split. For a direct comparison, both the full‐panel and restricted‐panel models were evaluated on the same 54 HY and *V. riparia* accessions. As shown in Table 3, restricting training to these two dominant groups reduced hair density RMSE from 0.559 to 0.480 in same‐year prediction and from 0.608 to 0.496 in cross year prediction, while tolerance accuracy increased from 63.0% to 68.5% and from 55.6% to 61.1%, respectively. Improvements for trichome density were smaller, with RMSE decreasing from 0.905 to 0.858 in same‐year prediction and from 0.914 to 0.900 in cross year prediction. These findings indicate that panel composition affects predictive performance, particularly for hair density. Although retaining the complete panel preserves potentially valuable wild species diversity, predictions for sparsely represented genetic groups should be interpreted cautiously and validated in larger, more balanced populations.

**TABLE 3:** Sensitivity analysis of linear‐transformer genotype‐to‐phenotype‐genotype‐to‐phenotype (LiT‐G2P) using the complete grapevine panel and the restricted panel.

Training panel	Test accessions	Scenario	Hair RMSE/MAE/Acc	Trichome RMSE/MAE / Acc
Full, *n* = 320	All, *n* = 64	Single‐year	0.609/0.477/60.9%	0.977/0.781/39.1%
Full, *n* = 320	All, *n* = 64	Cross‐year	0.684/0.533/56.2%	0.937/0.741/43.8%
Full, *n* = 320	HY+RI, *n* = 54	Single‐year	0.559/0.453/63.0%	0.905/0.752/40.7%
Full, *n* = 320	HY+RI, *n* = 54	Cross‐year	0.608/0.510/55.6%	0.914/0.737/40.7%
Restricted, *n* = 269	HY+RI, *n* = 54	Single‐year	0.480/0.401/68.5%	0.858/0.692/38.9%
Restricted, *n* = 269	HY+RI, *n* = 54	Cross‐year	0.496/0.401/61.1%	0.900/0.690/46.3%

Abbreviation: MAE, mean absolute error.

The SNP‐based narrow‐sense heritability estimates for grapevine leaf hair and trichome density are presented in Table 4. Marker‐based narrow‐sense heritability (*h* ^2^) estimates indicated moderate additive genetic control of leaf hair density, with *h* ^2^ values of 0.38 in 2023 and 0.50 in 2024. Trichome density showed substantially higher heritability, with estimates of 0.83 and 0.91, respectively. These results demonstrate that a meaningful proportion of phenotypic variation, particularly for trichome density, was associated with additive genomic differences among accessions. Nevertheless, these estimates do not exclude environmental or measurement effects because variation between the two vine replicates was not modeled separately. The lower predictive performance for trichome density despite its high *h* ^2^ may reflect the limited sample size, high marker dimensionality, and year‐dependent phenotypic variation rather than an absence of genetic signal. Broad‐sense heritability (*H* ^2^) was not estimated because the marker‐based model quantified additive genetic variance but did not separately partition dominance and variance.

**TABLE 4:** single‐nucleotide polymorphism (SNP)‐based narrow‐sense heritability estimates for grapevine leaf hair and trichome density.

Trait	Heritability (*h* ^2^)
Leaf hair 2022–2023 (baseline)	0.38
Leaf trichome 2022–2023 (baseline)	0.83
Leaf hair 2024 (target)	0.50
Leaf trichome 2024 (target)	0.91

Table 5 reports the top 5 SNPs most prioritized by the LiT‐G2P attention mechanism, providing an interpretable set of candidate makers that the model repeatedly relies on when predicting the target traits. Notably, the top‐ranked markers are concentrated on chromosomes #7 and #8, with three out of five on chromosome #8, suggesting that a subset of genomic regions may carry disproportionately informative signals for leaf hair and trichome variation in this diversity panel. The presence of two nearby variants at positions 18,542,650 and 18,542,656 on chromosome #8 further indicates a localized block or tightly linked region that the model considers important, consistent with the expectation that clustered SNPs can tag the same underlying causal variants. The allele summaries show that these prioritized SNPs span moderate to lower allele frequencies, implying that the model is not solely driven by common variants but can also utilize less frequent polymorphisms when they contribute predictive signal. While attention‐based prioritization does not establish causality by itself, these SNPs serve as a focused starting point for downstream validation, such as testing genotype‐stratified phenotype differences, mapping to nearby genes, or conducting targeted ablation analysis to quantify the change in prediction error.

**TABLE 5:** Top five informative single‐nucleotide polymorphisms (SNPs) prioritized by the linear‐transformer genotype‐to‐phenotype‐genotype‐to‐phenotype (LiT‐G2P) attention mechanism.

Index	#CHROM	POS	ID	REF	ALT	Info	Format
6729	8	21,324,057	SPN8_21324057	G	A	AC = 127; AN = 638	GT:DS
6617	8	18,542,656	SPN8_18542656	A	G	AC = 109; AN = 564	GT:DS
4943	7	1,394,640	SPN7_1394640	G	A	AC = 105; AN = 634	GT:DS
6616	8	18,542,650	SPN8_18542650	T	C	AC = 108; AN = 564	GT:DS
955	1	26,601,686	SPN1_26601686	A	G	AC = 31; AN = 624	GT:DS

Abbreviations: ALT, alternative allele; CHROM, chromosome; ID, marker identifier; INFO, additional information; POS, position; REF, reference allele.

The genotype‐stratified boxplots shown in Figure 5 provide an interpretable check of the top‐ranked SNPs by showing how trait values vary across genotype classes (0/1/2). Strongly associated SNPs are expected to stratify the phenotype by genotype class, producing clearly separated distributions including shifted medians and reduced overlap between genotype groups. According to the plot, it is observed that leaf hair density shows clearer genotype‐dependent separation than trichome density across the top SNPs. This stratification is consistent with the better predictive performance observed for leaf hair with higher accuracy, suggesting that leaf hair is driven by more readily learnable and stable genetic signal in this panel. Specifically, leaf hair density shows an overall trend for four out of the five SNPs, where genotype‐1 is associated with lower hair density relative to genotype‐0. The fifth SNP at position 26,601,686 displays a weaker reversed pattern, highlighting potential trait‐dependent effects. For trichome density, all five SNPs exhibit a consistent shift in distribution between genotype groups, with the genotype‐1 group generally showing higher trichome density than genotype‐0, suggesting a coherent allele‐dose effect for the alternate allele across markers. Overall, the repeated genotype‐dependent separation supports that the selected SNPs capture meaningful genetic signal aligned with phenotypic variation in the panel.

**FIGURE 5:**
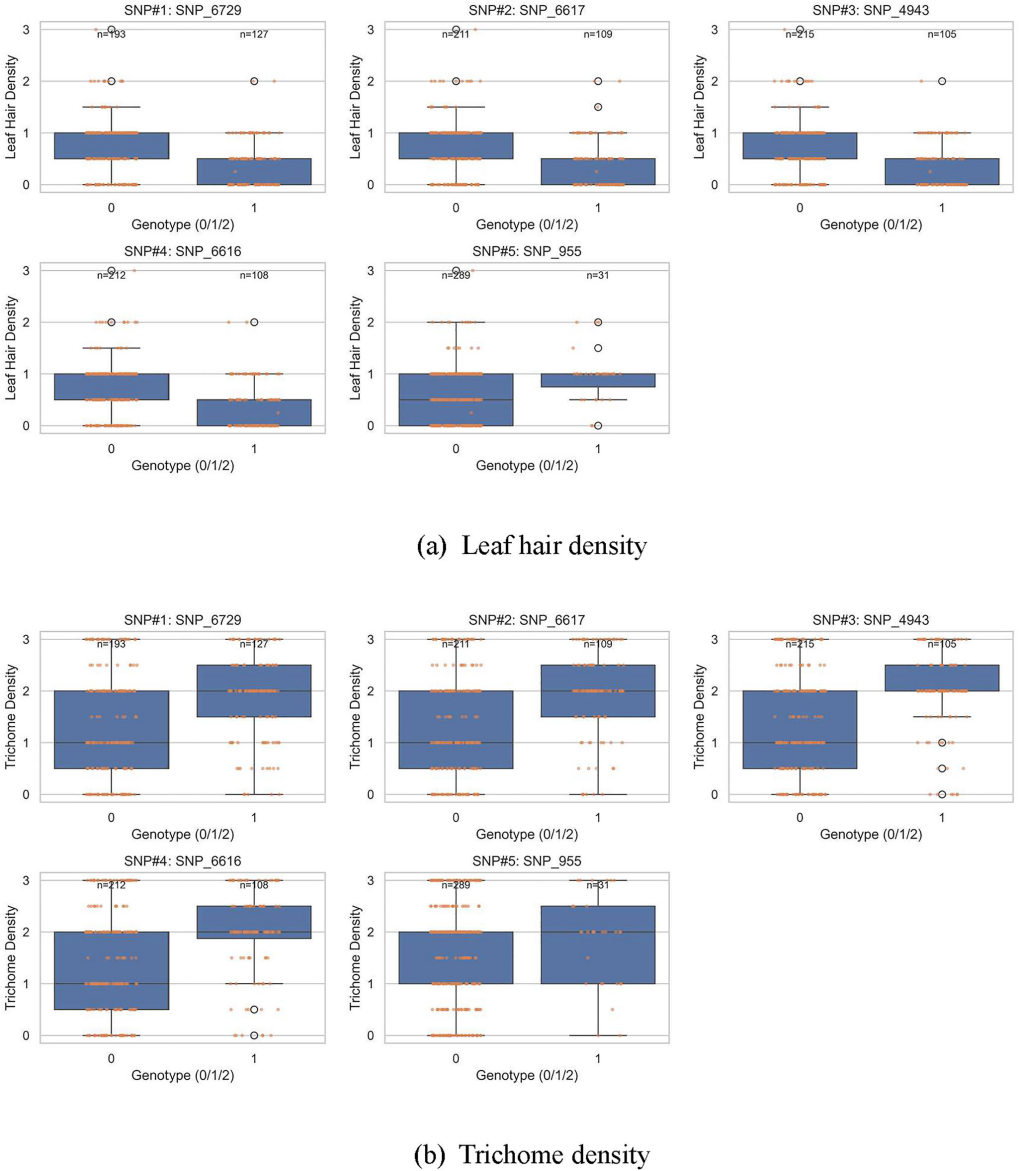
Genotype‐stratified (a) leaf hair density and (b) trichome density for the top five single‐nucleotide polymorphisms (SNPs).

Figure 6 presents the genome‐wide distribution of standardized trichome‐specific additive importance for all SNPs. The prioritized SNPs were distributed across chromosomes 2, 5, 10, 11, and 14. These markers differ from those identified using the earlier attention‐based ranking because Transformer attention summarizes shared nonlinear information across both traits and operates on 64‐SNP patches, whereas this analysis isolates individual SNP contributions to the trichome output of the additive branch and accounts for genotype variability. Consequently, the two rankings measure different components of LiT‐G2P and are not expected to coincide. The identified SNPs should be considered exploratory predictive markers rather than validated causal loci.

**FIGURE 6:**
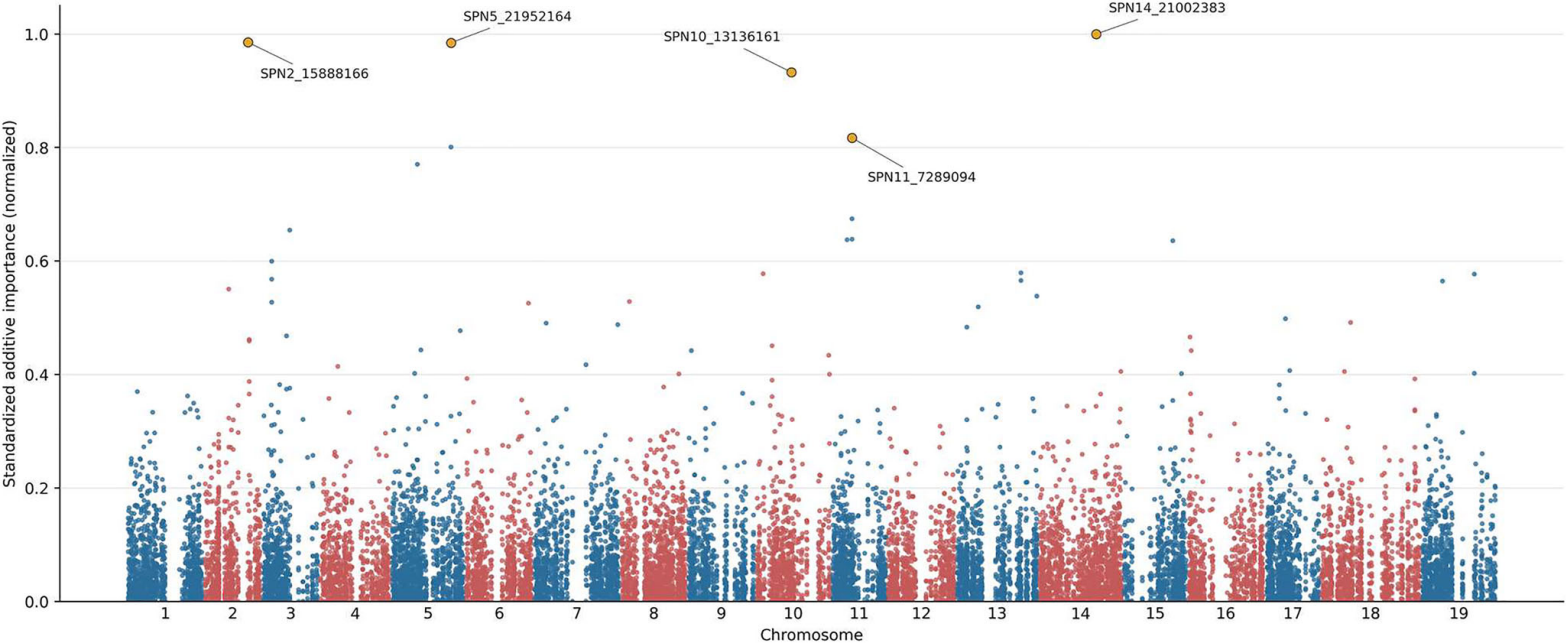
Distribution of linear‐transformer genotype‐to‐phenotype‐genotype‐to‐phenotype (LiT‐G2P) single‐nucleotide polymorphism (SNP) importance for trichome density across the grapevine chromosomes.

To complement prediction benchmarks with a well‐characterized biological signal, historical flower‐sex records from the USDA GRIN database were matched to 269 of the 320 genotyped accessions. An exploratory categorical marker‐phenotype screen across 15,388 SNPs, with missing calls excluded marker by marker, recovered the established flower sex‐determining region on chromosome 2. SNP at 4,949,616 bp occurred within the previously reported 4.907‐5.051 Mb interval and ranked third genome‐wide (*p* = 1.73 × 10^−19^). Moreover, 14 of the 40 markers within this interval occurred among the genome‐wide top 1%, representing significant enrichment (*p* = 9.36 × 10^−19^). This concordance provides an additional biological consistency check for genomic signal prioritization.

LiT‑G2P offers a practical and interpretable solution for improving SNP‑based prediction in grapevine. Integrating additive genetic effects with Transformer‑modeled interactions improved both the single‑year accuracy and cross‑year robustness of the model while providing biologically meaningful marker interpretation through attention‑based SNP prioritization. These capabilities make LiT‑G2P well‑suited for breeding pipelines, where earlier identification of elite accessions, reduced phenotyping burden, and greater stability across variable environments are critical for accelerating selection decisions. Despite these advantages, several limitations warrant further investigation. Although the overall SNP missingness was low, we imputed remaining missing calls using the major genotype, predominantly 0, to obtain a fixed‐dimensional SNP matrix for all accessions. This pragmatic strategy may introduce bias if missingness is non‐random or if rare‐allele patterns are affected. Future work will evaluate more principled imputation schemes and explicitly assess the sensitivity of predictive performance to alternative missing‐data treatments. Imbalanced genotype classes among high‑importance SNPs may restrict effect estimation, emphasizing the need for larger and more diverse panels. The improved hair‐density performance observed in the restricted HY and *V. riparia* analysis indicates that genetic‐group composition can influence prediction. Results for sparsely represented groups should therefore be considered exploratory until evaluated using larger and more balanced populations.

In addition, the study uses a single grapevine diversity panel and does not include external crop benchmarks. Consequently, conclusions should remain focused on grapevine rather than general method superiority. Extending to additional environments, years, management, and broader trait suites will better characterize robustness under various distribution shifts. Future work should explore additional modeling strategies that incorporate genotype‐by‐environment interactions to better capture environment‐dependent genetic effects. Pretraining the Transformer encoder on large multi‑crop genotype datasets also represents a promising direction toward developing a general‑purpose, foundation‑style SNP model that can be efficiently adapted across crops and traits rather than merely the grapevine breeding purpose.

## 4 CONCLUSION

This study presents LiT‐G2P, a HY G2P model that combines additive marker effects with Transformer‐based nonlinear interaction modeling. Within the evaluated panel of 320 grapevine accessions, LiT‐G2P showed favorable mean prediction performance for leaf hair and trichome density, particularly under cross‐year evaluation. However, the model‐based SNP importance analysis provides candidates for further investigation but does not establish causal associations. Evaluation using larger multi‐year and multi‐environment grapevine populations and independent biological validation will be necessary to confirm the robustness and breeding utility of the proposed approach. LiT‐G2P maintains higher tolerance accuracy and smaller performance degradation than competing methods, suggesting improved generalization under year‐to‐year shifts. Beyond predictive accuracy, LiT‐G2P provides a practical pathway toward interpretable AI‐assisted breeding and selection. The linear branch yields trait‐specific additive marker effects, while attention‐derived importance scores prioritize genomic regions and SNPs that contribute most to model predictions. Genotype‐stratified analysis of top SNPs reveals clear phenotype separation, supporting that the learned signals are biologically meaningful and consistent with the observed predictive performance. This provides targeted candidates for biological follow‐up. Nevertheless, cross‐year performance remains limited by environmental variability and phenotyping noise, which highlights the demand for larger multi‐year grapevine breeding data and explicit modeling of genotype‐by‐environment effects. Future work will extend LiT‐G2P to multi‐environment training and domain‐shift regularization and validate prioritized SNPs using genotype‐stratified analysis on external populations.

## AUTHOR CONTRIBUTIONS

**Yibin Wang**: Conceptualization; data curation; formal analysis; investigation; methodology; software; validation; visualization; writing—original draft. **Murukarthick Jayakodi**: Conceptualization; data curation; validation; writing—review and editing. **Silvas Kirubakaran**: Conceptualization; data curation; validation; writing—review and editing. **Ambika Chandra**: Conceptualization; writing—review and editing. **Azlan Zahid**: Conceptualization; funding acquisition; project administration; resources; supervision; writing—review and editing.

## CONFLICT OF INTEREST STATEMENT

The authors declare no conflicts of interest.

## Supporting information



Supplementary Material

## Data Availability

All data generated in this study are provided in Table S1. This dataset includes accession‐level measurements derived from fully matured, physiologically active third leaves sampled from two vines per accession. The botanical nomenclature of all accessions evaluated in this study can be queried in the GRIN‐Global Database (https://www.ars‐grin.gov/). All data necessary to interpret, evaluate, and reproduce the findings of this study are provided within the article and its Supporting Information.
